# Publisher Correction: Optoplasmonic characterisation of reversible disulfide interactions at single thiol sites in the attomolar regime

**DOI:** 10.1038/s41467-020-16611-z

**Published:** 2020-06-09

**Authors:** Serge Vincent, Sivaraman Subramanian, Frank Vollmer

**Affiliations:** Living Systems Institute, School of Physics, University of Exeter, Exeter, EX4 4QD UK

**Keywords:** Chemical physics, Characterization and analytical techniques, Imaging and sensing

Correction to: *Nature Communications* 10.1038/s41467-020-15822-8, published online 27 April 2020.

In the original version of this Article “5,5′-dithiobis-(2-nitrobenzoic acid)” was incorrectly written as “5,5′-dithiobis-(2-nitrobenzoic) acid”, and in Eq. (1) the upper limit of the summation in the denominator “n” was incorrectly written as “T”.

This has been corrected in both the PDF and HTML versions of the Article.

