# Supplementary material for: Optoplasmonic characterisation of reversible disulfide interactions at single thiol sites in the attomolar regime
Source: Nat Commun. 2020 Apr 27;11:2043. doi: 10.1038/s41467-020-15822-8 (PMC7184569; doi:10.1038/s41467-020-15822-8)
Supplement: Supplementary file 1 — Supplementary Information [file 41467_2020_15822_MOESM1_ESM.pdf]

Supplementary Information

Optoplasmonic Characterisation of Reversible Disulfide Interactions at Single Thiol Sites in the Attomolar Regime

Vincent *et al.*

Supplementary Methods 1: Single-Molecule Data Analysis

Empirical determination of spike and step signal properties is in compliance with selection criteria. Reference measurements are conducted before each step of an experiment to calculate noise floor figures, such as the background's standard deviation σ. Following the detrending of the resonance trace via a Savitzky-Golay filter, the shift value, time stamp, and time duration of transient signals (in either the resonant wavelength or linewidth) are extracted if fluctuations are smaller than -3σ or larger than 3σ. Spike packets are grouped when their time stamps lie within a specified time interval, e.g. neighbouring fluctuations less than 50 ms apart. The time scale for single-molecule interactions with the plasmonic nanoantennea is often shorter than our system's time resolution – a disparity that is crucial when identifying patterns. Steps in the resonance trace present themselves as discontinuous jumps that correlate to the sub-ms transport of analytes to the hybrid resonator, or simply molecular activity once an analyte is bound inside a plasmonic hotspot. The temporal axis thus provides a basis for comparison between signal states when discerning different interaction events that perturb the optoplasmonic sensor.

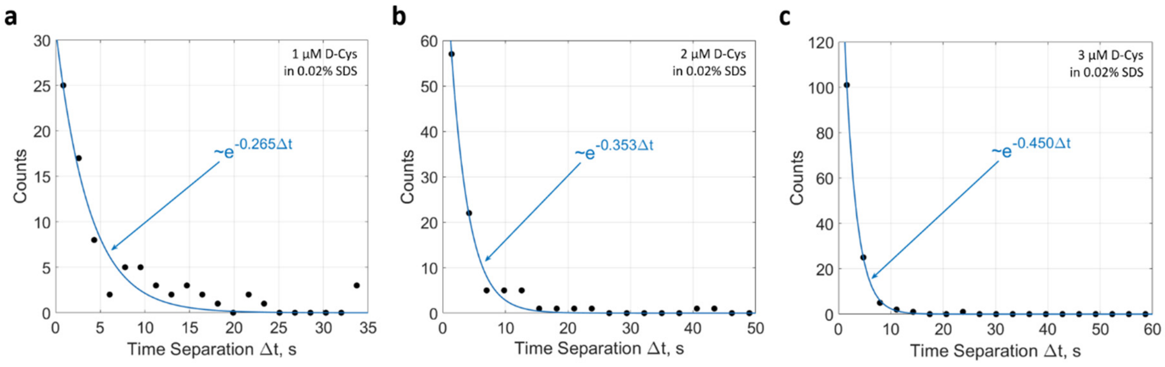

Supplementary Figure 1 | Single D-cysteine statistics. Event time separations at **a,** 1 µM, **b,** 2 µM, and **c,** 3 µM are Poisson-distributed and the mean event rates are those used for **Fig. 1d** of the main manuscript.

Statistical assessment of the trace data is carried out by histogram plotting. If 1) each instance of an event is stochastically independent to every other occurrence of that event and 2) the rate scales linearly with reactant concentration, we deem the interaction associated with single molecules. Under this assertion the Poissonian probability distribution for k events to transpire in a time interval Δt should have the form:

$$P(k, R, \Delta t) = \frac{(R\Delta t)^k}{k!} e^{-R\Delta t} \quad (1)$$

where R is the mean event rate. **Suppl. Fig. 1** above displays this behaviour for ramping concentration and the implied convergence to a Poisson distribution as events accumulate. Signal extent and duration similarly fall within probability distributions to be approximated by bin discretisation. For all analyses of resonance parameters, we apply the Freedman-Diaconis rule to calculate histogram bin widths.

Supplementary Methods 2: Microcavity Surface Preparation

Earlier functionalisation techniques [1] for the optoplasmonic resonator incorporated electrostatic adsorption of gold nanorods (NRs) to a fused silica surface. This was a standard even for observing kinetics of enzymes on immobilised DNA [2]. At a basic pH above 7, however, the NRs' cetyltrimethylammonium bromide coating destabilises [3] as to cause further noise and eventual desorption of the NRs. Going forward, we sought suitable alternatives to electrostatic adsorption which were proven resistant to environmental pH. One option was to bind the plasmonic NRs to the amine groups of an amine-functionalised silica. This wet-chemical procedure is initiated immediately after CO_2 laser reflow of the microsphere by full immersion into a 1% (v/v) solution of 97% N-[3-(Trimethoxysilyl)propyl]ethylenediamine (in ultrapure water) for 45 s.

After aminosilanising the silica microcavity, the gold NRs are immobilised and detected as in **Suppl. Fig. 2a**. Depending on the exact shape of these NRs, resonance shifts may be positive, negative, or zero depending on where the localised surface plasmon resonance lies with respect to the excitation wavelength. The absorption/scattering losses experienced by the whispering-gallery mode from bound gold NRs is substantial, hence broadening in the linewidth on the order of 1-10 fm is common as long as the nanoparticles are close to the equator and overlap with the evanescent field. Finally, primary amines of the partial aminosilane coating are modified with methyl-terminated, N-hydroxysuccinimide (NHS) ester-activated polyethylene glycol (PEG) to neutralise surface charges and passivate the microcavity surface. Bulk binding is measured in **Suppl. Fig. 2b**, as is single methyl-NHS-PEG binding around the gold NRs in **Suppl. Fig. 2c** as these events far outnumber the available plasmonic sensing sites. Two classes of signal appear here: 1) frequent redshifts in the resonant wavelength and 2) redshifts accompanied by linewidth narrowing or broadening.

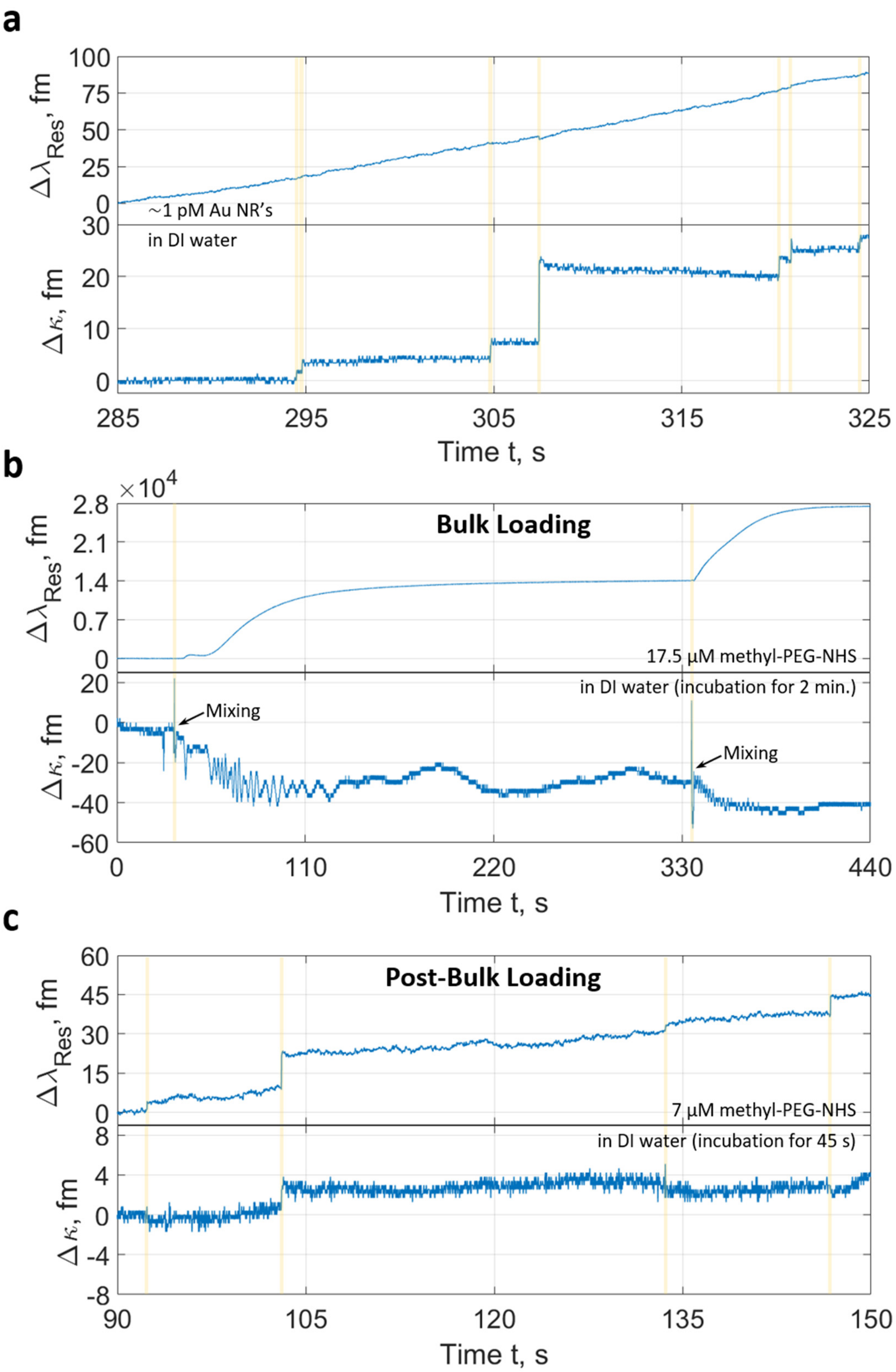

Supplementary Figure 2 | Surface functionalisation of the glass microsphere. a, Gold NR deposition onto the microcavity via binding to the amine terminals of an aminosilane multilayer. **b,** Bulk capping of surplus amines of the aminosilane multilayer with methyl-PEG-NHS ester compounds. Saturation occurs in accordance with the reactive sensing principle [4]. **c,** Single methyl-NHS-PEG binding steps in proximity to plasmonic detection sites. Traces are shown in their raw form and so environmental noise, such as thermal drift, is visible.

Supplementary Notes 1: Additional Aminothiol Control

The functional groups of cysteine have been modelled in the past with density functional theory as to gauge how these interact with gold atom clusters [5]. Amine-gold anchoring was concluded therein to be energetically favourable for complex stabilisation and that lone pair electrons are transferred to the antibonding orbitals of gold. Peptide/amide bonds can be formed between carboxyl and amine groups through dehydration synthesis and carboxyl group interactions with gold clusters have been found to be non-negligible for some molecular charge states [6]. To eliminate this ambiguity and illustrate the importance of implementing reducing agents, we studied dimer formation/cleavage solely with cysteamine in a basic buffer.

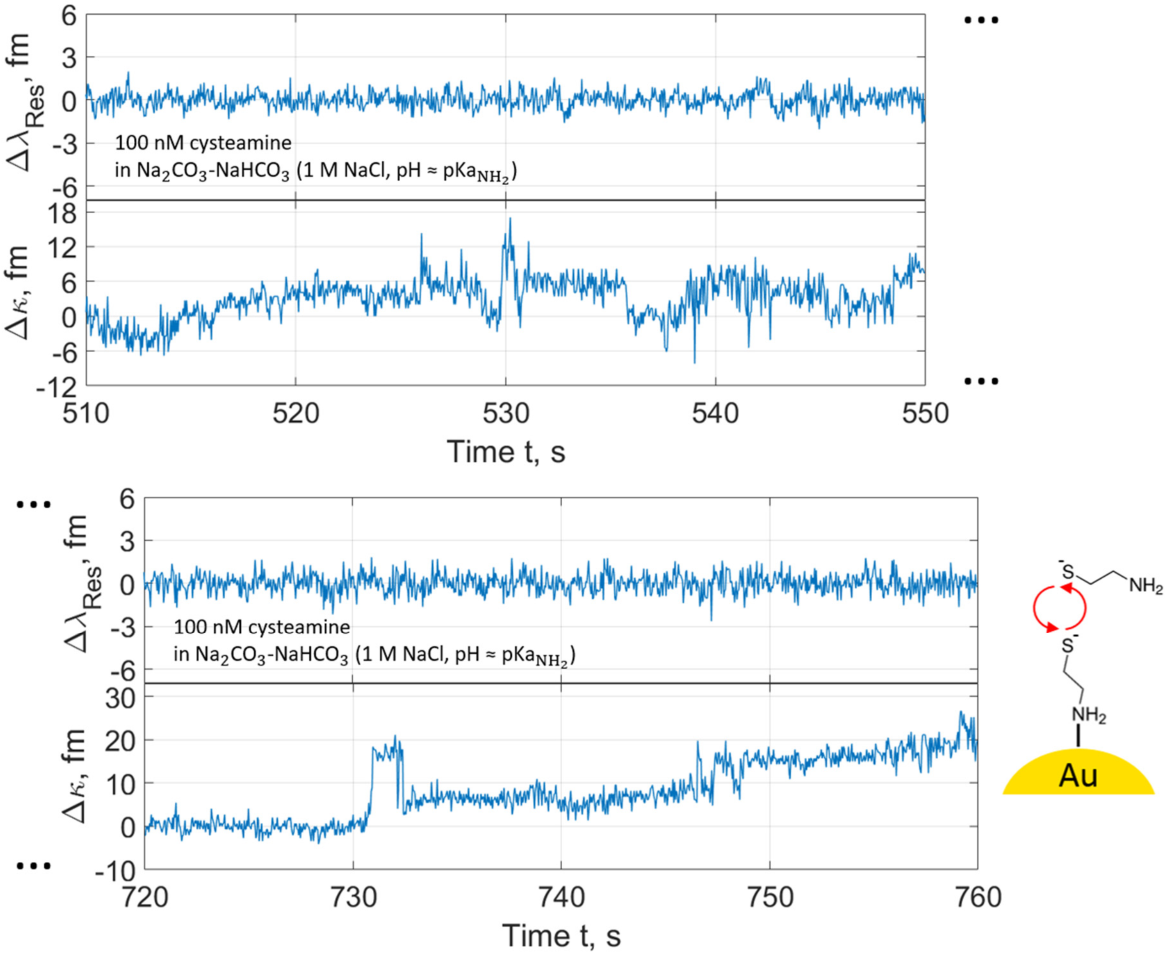

Supplementary Figure 3 | Sample trace for cysteamine disulfide reactions. Signal levels in κ vary widely in the absence of tris(2-carboxyethyl)phosphine. The vulnerability of one of the two sulfur atoms in a disulfide bond to a nucleophilic thiolate attack, whether the dimer is immobilised onto gold or not, provides reaction bi-directionality and stochastic population of the detection sites with bound dimers. The optoplasmonic linewidth thus walks off during the trace segment.

Suppl. Fig. 3 depicts short bursts of linewidth oscillations for 100 nM cysteamine akin to the pulse train of **Fig. 5a,b**. These sporadic disulfide binding/unbinding events are reproducible at high analyte concentration without tris(2-carboxyethyl)phosphine, albeit step transitions are unpredictable with increased dwell time variance. Negatively charged thiolates in solution, like dithiothreitol, act as reducing agents by thiol-disulfide exchange with the outermost aminothiol of a bound disulfide. This reaction pathway could leave bound thiolate linkers and close a redox cycle, translating to pulse outliers in the resonant linewidth channel (i.e. an amalgamation of the events expounded in **Suppl. Fig. 4**). Spike activity is also interspersed throughout the trace in agreement with the pH value being close to the $\mathrm{pKa}_{\mathrm{NH_2}}$, as a proportion of the cysteamines' amines are protonated as in **Fig. 4b**. The resonant wavelength does not drastically change in this control and therefore we interpret that cysteamine, in this case, seldom interacts with the gold directly.

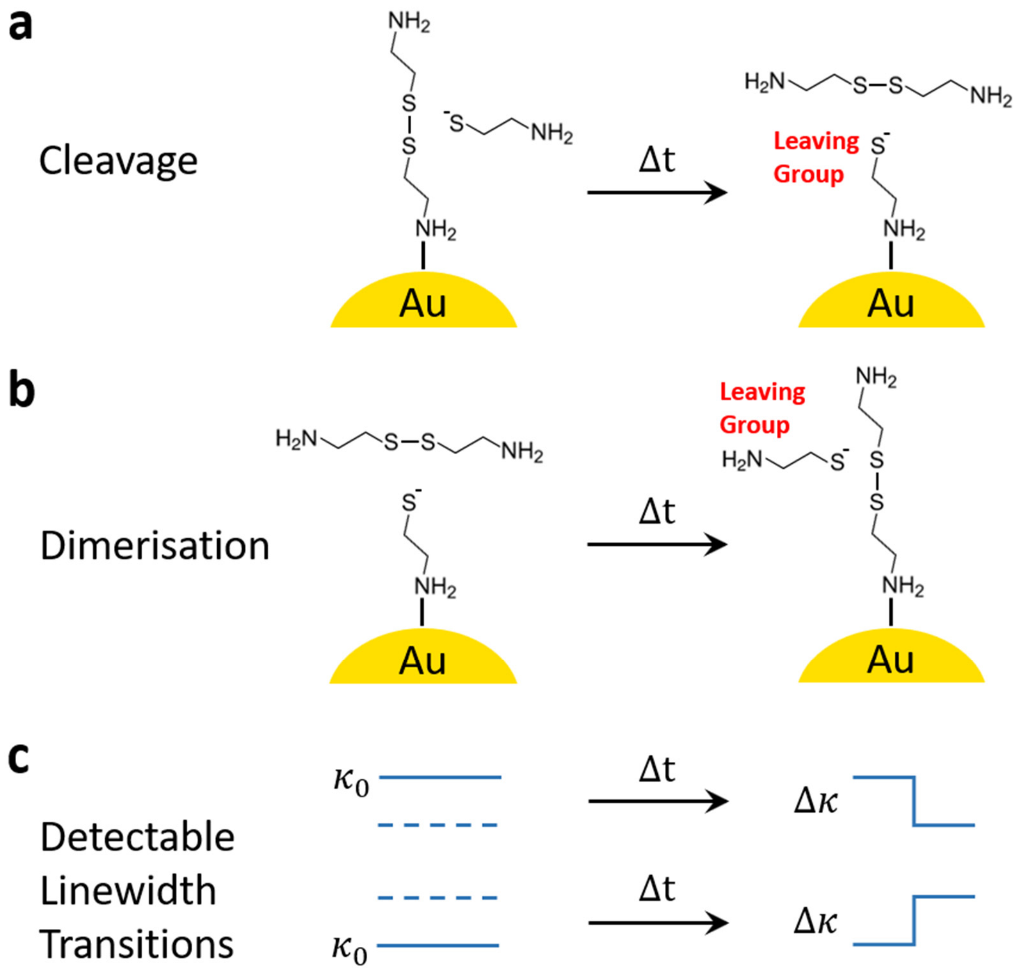

Supplementary Figure 4 | Thiol-disulfide exchange pathways. The leaving group may be discriminated based on the optoplasmonic linewidth signal post-disulfide exchange, i.e. a discrete step signifies **a,** a single bound dimer that is cleaved or **b,** a bound monomer that is dimerised. Further deductions in **c** require knowledge of the initial molecule state as binding can either narrow or broaden the linewidth (cf. **Fig. 2** of the main manuscript).

Supplementary Notes 2: Dilution Uncertainties

As this work involves trace element analysis nearing extreme concentration limits, we must consider propagating experimental uncertainties. Serial dilutions will amplify the absolute error Δc_i at each concentration stage c_i:

$$\frac{\Delta c_i}{c_i} = \sqrt{\left(\frac{\Delta V_i}{V_i}\right)^2 + \left(\frac{\Delta V_{\mathrm{T},i}}{V_{\mathrm{T},i}}\right)^2} \tag{2}$$

wherein we dilute a volume of dissolved analyte V_i, with absolute error ΔV_i, in a total volume $V_{\mathrm{T},i}$, with absolute error $\Delta V_{\mathrm{T},i}$. The variable pipettes for dispensing liquids in this protocol are *Gilson* P2L, P20L, and P1000L assemblies with respective maximal errors of ±0.030 µL for 1.0 µL, ±0.11 µL for 10 µL, and ±8.1 µL for 1000 µL. In all cases, the stock solution concentration is on the order of 1 mM. The concentration uncertainty then attained in the process of diluting to 100 aM, by diluting three times by a factor of 10^3 and once by a factor of 10^2, becomes:

$$\Delta c_{100\mathrm{aM}} = (100\ \mathrm{aM})\sqrt{3\left(\frac{\pm 0.030\ \mathrm{\mu L}}{1.0\ \mathrm{\mu L}}\right)^2 + \left(\frac{\pm 0.11\ \mathrm{\mu L}}{10\ \mathrm{\mu L}}\right)^2 + 4\left(\frac{\pm 8.1\ \mathrm{\mu L}}{1000\ \mathrm{\mu L}}\right)^2}$$

$$= \pm 5.6\ \mathrm{aM}$$

This estimated uncertainty, which is less than an order of magnitude, delineates the worst case deviation from expected concentrations for single-molecule detection when approaching 100 aM. The main manuscript omits such deviations for brevity purposes; notwithstanding, it is clear here that all stated concentrations inherent at most a 5.6% relative error.

Supplementary References

[1] Baaske, M. D., Foreman, M. R. & Vollmer, F. Single-molecule nucleic acid interactions monitored on a label-free microcavity biosensor platform. *Nat. Nanotechnol.* **9**, 933-939 (2014).

[2] Kim, E., Baaske, M. D., Schuldes, I., Wilsch, P. S. & Vollmer, F. Label-free optical detection of single enzyme-reactant reactions and associated conformational changes. *Sci. Adv.* **3**, doi: 10.1126/sciadv.1603044 (2017).

[3] Ding, H., Yong, K.-T., Roy, I., Pudavar, H. E., Law, W. C., Bergey, E. J. & Prasad, P. N. Gold Nanorods Coated with Multilayer Polyelectrolyte as Contrast Agents for Multimodal Imaging. *J. Phys. Chem. C* **111**, 12552-12557 (2007).

[4] Arnold, S., Khoshsima, M., Teraoka, I., Holler, S. & Vollmer, F. Shift of whispering-gallery modes in microspheres by protein adsorption. *Opt. Lett.* **28**, 272-274 (2003).

[5] Xie, H.-J., Lei, Q.-F. & Fang, W.-J. Intermolecular interactions between gold clusters and selected amino acids cysteine and glycine: a DFT study. *J. Mol. Model.* **18**, 645-652 (2011).

[6] Pakiari, A. H. & Jamshidi, Z. Interaction of Amino Acids with Gold and Silver Clusters. *J. Phys. Chem. A* **111**, 4391-4396 (2007).
